# Coping strategies and adherence in people with mood disorder: a cross-sectional study

**DOI:** 10.3389/fpsyt.2024.1400951

**Published:** 2024-05-21

**Authors:** Alicja Jeżuchowska, Daria Schneider-Matyka, Kamila Rachubińska, Artur Reginia, Mariusz Panczyk, Dorota Ćwiek, Elżbieta Grochans, Anna Maria Cybulska

**Affiliations:** ^1^ Department of Clinical Nursing, Faculty of Health Sciences, Wroclaw Medical University, Wroclaw, Poland; ^2^ Department of Nursing, Faculty of Health Sciences, Pomeranian Medical University in Szczecin, Szczecin, Poland; ^3^ Department of Psychiatry, Pomeranian Medical University, Szczecin, Poland; ^4^ Department of Education and Research in Health Sciences, Faculty of Health Sciences, Medical University of Warsaw, Warsaw, Poland; ^5^ Department of Obstetrics and Pathology of Pregnancy, Pomeranian Medical University in Szczecin, Szczecin, Poland

**Keywords:** adherence, coping strategies, stress, mood disorder, quality of life

## Abstract

**Introduction:**

Non-adherence to treatment recommendations is a significant problem, as it contributes to the progression of the disease and to the exacerbation of distressing symptoms. Failure to cope with the disease and elevated levels of stress, in turn, influence the choice of strategy for coping with a difficult situation, and thus adherence to recommendations.

**Objectives:**

The purpose of our study was to evaluate the impact of the subjects’ stress coping styles on therapeutic adherence, life satisfaction, disease acceptance and quality of life (QoL) in people with mood disorders.

**Methods:**

This survey-based study included 102 respondents diagnosed with mood disorders, living in the West Pomeranian Voivodeship. It was performed using the sociodemographic questionnaire and standardized tools: The Coping Inventory for Stressful Situations (CISS), The Satisfaction with Life Scale (SWLS), The Short Form-36 (SF-36) Health Survey, The Adherence to Refills and Medication Scale (ARMS), and The Acceptance of Illness Scale (AIS).

**Results:**

Some 47.06% of the respondents suffered from depressive disorders, while 34.31% had depression or mixed anxiety disorder. Patients who made greater use of an emotion-focused style were found to have significantly lower life satisfaction than other patients. Moreover, this style was related to such SF-36 domains as general health, social functioning, role emotional, vitality, and mental health, as well as to physical component summary (PCS) and mental component summary (MCS).

**Conclusion:**

Treatment non-adherence is a serious challenge in the treatment of patients with mood disorders. Individuals who do not adequately follow treatment recommendations often resort to alternative activities as a mechanism for coping with difficult situations. Patients who predominantly adopt an emotion-oriented coping style tend to experience lower life satisfaction and greater difficulty accepting their condition compared to their peers. Conversely, patients who adopt a task-oriented coping style report better quality of life than those who rely on emotion-oriented coping or alternative activities.

## 1 Introduction

Mental disorders constitute a global public health challenge, with almost 450 million people suffering from them worldwide (1). Mood disorders are among the most prevalent and debilitating mental conditions affecting world population. They are the second most common category of mental disorders after anxiety disorders, and contribute to an increase in mortality among those affected (2). Mental disorders affect individuals’ current functioning, as well as their quality of life (QoL) and life satisfaction (3). The symptomatology of mood disorders is very rich and varied. Individual symptoms occur with different frequency. Primary symptoms include depressed mood, a lack of energy, objectively visible psychomotor retardation, anhedonia, and a lack of self-esteem (4). Mental disorders are related to individual factors, as well as social support, culture, social protection, standard of living, and other environmental issues (5). Therefore, treatment adherence is important, but a challenge in treating serious mental disorders (6).

Adherence to treatment recommendations is defined as the patient’s acceptance of and compliance with basic health and treatment suggestions; this definition covers various aspects, such as access to treatment, taking medications, and understanding further advice (7–9). The WHO defines medication non-adherence as “a case in which a person’s behavior while taking medication does not conform to the established recommendations of medical personnel” (10). The term “treatment adherence” is therefore broader than “medication adherence”, which refers only to prescribed medications. A review of the literature indicates that treatment adherence is difficult to achieve, and is an obstacle to achieving good clinical outcomes in people with severe mental disorders (11). Patients with serious psychiatric problems are most likely to fail to adhere to medication due to poor reasoning and lack of knowledge about their illness and treatment (8, 12, 13). In turn, non-compliance with taking psychotropic drugs can lead to exacerbation of the disease, reduce the effectiveness of treatment or make the patient less amenable to subsequent treatment. Other consequences include: rehospitalization, worse QoL, recurrence of symptoms, and increased suicide rates (14–16). Since non-adherence is recognized to elevate the risk of unfavorable clinical outcomes, patient-specific non-adherence risk should be evaluated, and individuals at high risk for non-adherence should be closely monitored (17). However, it should be mentioned that some researchers have not found a direct positive effect of medication adherence on the functioning of patients with mood disorders (18).

Stress coping involves cognitive and behavioral strategies used by people facing stressful situations and life events (19). The choice of stress coping strategies has an impact on how patients perceive the symptoms they experience, and how they deal with them. Coping can be defined as thoughts and behaviors that are used to handle internal and external demands of situations perceived as stressful (20). A review of the literature indicates that coping styles are closely related to an individual’s health, and is a mechanism in between stress and disease. If patients’ coping is not effective and properly managed, it may have a negative impact on both their QoL and mental state (21). Active coping strategies have been shown to improve psychosocial functioning, while maladaptive strategies (e.g. pondering on negative states) have been linked to increased depression (22). Apart from influencing the course of the disease, various coping strategies (e.g., low levels of acceptance and high levels of denial) are associated with poor adherence to treatment recommendations (23). Denial is defined as a lack of belief in the existence of a stressor or an attempt to recognize the stressor as untrue, while acceptance is the awareness that a given stressful situation is real and should be dealt with (24). Acceptance is associated with many benefits, for example it makes it easier to cope with the disease and is a key predictor of recovery (24, 25). A review of the literature indicates that patients with mood disorders tend to use maladaptive or emotion-oriented strategies (26), which may affect their QoL, perception of their own illness, and adherence to treatment recommendations.

According to recent reports, so far no factors have been identified that affect treatment adherence among patients with mood disorders. There are a number of potential variables that may affect adherence to treatment recommendations. These may be related to the patient (socioeconomic characteristics, perceptions, beliefs), the doctor (doctor-patient relationship), the treatment (efficacy, side effects, drug dose, number of pills, number of medications), and the type of disorder (severity of illness). Each of these risk factors can affect adherence and interact with other factors. Therefore, it is important to examine the variables that increase or decrease adherence. The main objective of our study was to assess the impact of the subjects’ stress coping styles on their therapeutic adherence, life satisfaction, disease acceptance, and QoL.

## 2 Materials and methods

### 2.1 Settings and design

The study recruited 150 people diagnosed with mood disorders, living in the West Pomeranian Voivodeship. The inclusion criteria were: age over 18, confirmed clinical mood disorders (depression, bipolar disorder, depressive disorder, and mixed anxiety disorder), consent to participate in the study, and completion of the entire questionnaire. The exclusion criteria were: age below 18 years, lack of consent to participate in the study, no mood disorders, and failure to complete the entire questionnaire.

The study was conducted in accordance with the Declaration of Helsinki after obtaining approval from the Bioethics Committee of the Pomeranian Medical University in Szczecin (KB-0012/153/17). It is part of a larger project that aims to identify factors affecting treatment adherence, life satisfaction, acceptance of the disease, and QoL in people with mood disorders.

This survey-based study was performed using a questionnaire technique, and the traditional method of distributing paper copies of the questionnaires was employed. After obtaining approval for the study, the previously prepared questionnaires were administered to individuals. The respondents were informed about the study’s objectives and the opportunity to ask questions and receive comprehensive answers. They also learned that participation in the study was anonymous and that they could opt out at any stage. Then, they provided consent to take part in the project. Ultimately, 102 people (68%) were included.

### 2.2 Research tools

Data collection was carried out using the sociodemographic questionnaire containing a series of questions on basic sociodemographic data (age, sex, marital status, education, place of residence, employment status), and medical data (type of disorder, duration of illness, time of taking medications, number of medications used, awareness of the need to attend medical appointments). Additionally, the following standardized research tools were applied:

• The Coping Inventory for Stressful Situations (CISS) is a 48-item self-report questionnaire that measures three types of stress coping strategies, namely: task-oriented coping (TOC), emotion-oriented coping (EOC), and avoidance-oriented coping (AOC). The last of these styles takes two forms: distraction (engaging in alternative activities), and social diversion (seeking social contact). The results are converted into sten norms, with 1-3 sten scores regarded as low results, 4-7 sten scores—average results, and 8-10 sten scores—high results. Higher scores indicate increased utilization of a specific coping strategy. The value of Cronbach’s alpha ranges from 0.7 to 0.88 depending on the subscale (27, 28).
• The Satisfaction with Life Scale (SWLS)―is a tool that assesses the level of satisfaction with life. It contains 5 statements rated on a seven-point Likert scale, where 1 means *I strongly disagree*, and 7―*I strongly agree*. The total score ranges from 5-35 points― the higher the score, the higher the satisfaction with life. When interpreting the results, reference should be made to the sten norms: 1-4 sten―low score, 5-6 sten―average score, 7-10 sten―high score. The value of Cronbach’s alpha was 0.87 (29).
• The Short Form-36 (SF-36) Health Survey is one of the most frequently used health-related quality of life (HRQoL) measures. It consists of 35 items divided into eight *health* domains, namely: physical function (PF) (10 items), role physical (RP) (4 items), bodily pain (BP) (2 items), general health (GH) (5 items), vitality (VT) (4 items), social functioning (SF) (2 items), role emotional (RE) (3 items), and mental health (MH) (5 items). Some items are rated on a 3-point scale, while others on a 5-point scale. The domains can be combined into two summary measures:
  - physical component summary (PCS)—calculated as the sum of PF, RP, BP, and GH scores,
  - mental component summary (MCS)—calculated as the sum of RE, SF, MH, and VT scores.
  - The 36^th^ item—self-reported *health transition* (HT)—asks about health change, and does not contribute to the domain or summary scores.
  - The quality of life index is the sum of points obtained in all eight scales, and reflects the overall HRQoL. The possible scores range from 0 to 100—the higher the score, the better the QoL (30).
• The Adherence to Refills and Medication Scale (ARMS) consists of 12 questions divided into two subscales: the first 8 questions are about medication adherence, and the next 4 questions are about prescription adherence. Each answer is scored on a 4-point Likert scale, where 1 = *never*, and 4 = *most of the time*. The total score reflects the general level of adherence, with a low score indicating better adherence to the recommendations (31).
• The Acceptance of Illness Scale (AIS) is used to assess the degree of acceptance of a disease―the greater the acceptance, the better the adaptation and the lesser the psychological discomfort. The AIS contains eight statements rated on a five-point Likert scale, where 1 denotes poor adaptation to a disease, and 5 its full acceptance. Low scores (0–29) indicate a lack of acceptance and poor adaptation to a disease, and strong mental discomfort. High scores (35–40) indicate acceptance of the disease, manifesting as a lack of negative emotions associated with it. The total score is a sum of all points, and can range from 8 to 40, reflecting the overall degree of acceptance of the disease (32).

### 2.4 Statistical analysis

Quantitative and categorical variables were characterized using descriptive statistics methods. For quantitative variables, we determined: measures of central tendency (M—mean, Mdn—median), measures of variability (SD—standard deviation, IQR/2— interquartile range, CV—coefficient of variation). For categorical variables, measures of structure were determined: number (n) and frequency (%). The results of standardized psychometric measurements were calculated in accordance with the principles described by their creators, and transformed into a sten scale using norms for the Polish general population.

Classical statistics based on null hypothesis testing was used for statistical inference. The effects of selected sociodemographic and medical variables, the severity of depression symptoms, ways of coping with stress, and selected personality traits on life satisfaction (according to the SWLS), QoL (according to the SF-36), adherence (according to the ARMS) and acceptance of the disease (according to the AIS) were analyzed using regression models. Multivariate linear regression models with least-squares parameter estimation were tested. The multicollinearity of independent variables was checked by determining the tolerance coefficient to avoid model redundancy.

Categorical variables were coded using a *sigma-constraint method* (quasi-experimental). All predictors were entered into the model simultaneously. Stepwise progressive introduction of predictors was used to build a model of determinants that explained the variance of the dependent variable to the greatest extent. The degree of overall explained variance of the dependent variable was estimated by determining the adjusted R^2^ value. For each predictor, the unstandardized (b) and standardized (β_stand._) regression coefficients along with the 95% confidence interval (95% CI) were determined.

A default statistical significance level of 0.05 was assumed for all analyses. Calculations were performed using the Statistica v. 13.3 software (TIBCO Software Inc., Palo Alto, California, USA).

## 3 Results

### 3.1 Brief characteristics of the respondents

The study included 102 respondents diagnosed with mood disorders, the majority of whom were women (91.18%), people in a relationship (64.71%), those living in cities with over 100,000 inhabitants (48.04%), and those employed (59.80%). Some 47.06% of the respondents suffered from depressive disorders, while 34.31% had depression or mixed anxiety disorder. The remaining respondents had bipolar disorder (11.76%) or other types of disorders (6.86%). The duration of the disorder ranged from 1 to 36 years (Me = 7 years) (**Table 1**).

**Table 1:** Characteristics of the study group by sociodemographic status.

Variable		n	%
Sex	woman	93	91.18
	man	9	8.82
Marital status	relationship	66	64.71
	single	36	35.29
Education	primary/vocational	15	14.71
	secondary	28	27.45
	third-level	59	57.84
Place of residence	rural areas	22	21.57
	a city of up to 100,000 people	31	30.39
	a city of over 100,000 people	49	48.04
Employment status	employed	61	59.80
	unemployed	41	40.20

Frequency (N), percentage (%)

### 3.2 Analysis of variable values

The study analyzed stress coping styles according to the CISS, life satisfaction according to the SWLS, QoL according to the SF-36, adherence according to the ARMS, and acceptance of the disease according to the AIS.

**Table 2** shows the ways of coping with stress according to the CISS. Based on the collected data, it was shown that the subjects obtained the lowest average score for task-oriented coping (TOC) and the highest for avoidance-oriented coping (EOC). For life satisfaction (SWLS), adherence (ARMS), and acceptance of illness (AIS), the respondents scored 15.8 points, 19.13 points, and 23.47 points, respectively.

**Table 2:** The subjects’ stress coping styles according to the CISS, life satisfaction according to the SWLS, adherence according to the ARMS, acceptance of illness according to the AIS, and QoL according to the SF-36.

Variable		M	SD	Me	min	max
CISS (sten score)	Task-oriented coping	4.00	1.97	4.0	1.0	10.0
	Emotion-oriented coping	6.93	2.31	7.0	1.0	10.0
	Avoidance-oriented coping	5.81	1.97	6.0	1.0	10.0
	Distraction (engaging in alternative activities)	6.79	1.87	7.0	2.0	10.0
	Social diversion (seeking social contact)	4.33	2.34	5.0	1.0	10.0
Satisfaction with Life Scale (SWLS)		15.80	6.65	16.0	5.0	34.0
Adherence to Refills and Medication Scale (ARMS)		19.13	5.66	18.0	12.0	35.0
Acceptance of Illness Scale (AIS)		23.47	7.97	24.0	8.0	40.0
SF-36	Total score	23.47	7.97	24.0	8.0	40.0
	Physical function (PF)	81.07	18.98	85.0	20.0	100.0
	Role physical (RP)	54.00	25.21	50.0	0.0	100.0
	Bodily pain (BP)	61.94	23.05	60.0	10.0	100.0
	General health (GH)	37.28	13.64	36.0	8.0	72.0
	Vitality (VT)	38.74	16.11	35.0	10.0	90.0
	Social functioning (SF)	45.63	26.30	50.0	0.0	100.0
	Role emotional (RE)	46.68	27.05	50.0	0.0	100.0
	Mental health (MH)	44.04	15.38	44.0	16.0	88.0
	Health transition (HT)	49.03	31.30	50.0	0.0	100.0
	Physical component summary (PCS)	66.56	14.63	68.2	22.7	100.0
	Mental component summary (MCS)	43.09	16.52	40.0	9.2	92.3

M, mean; SD, standard deviation; Me, median.

The highest average QoL scores (SF-36) were obtained for the domains of physical function (PF) (81.07 points) and bodily pain (BP) (61.94 points), and for physical component summary (PCS) (66.56 points). The lowest average QoL scores were achieved for vitality (VT) (38.74 points) and general health (GH) (37.28 points) (**Table 2**).

The study analyzed the impact of stress coping styles according to the CISS on selected variables such as life satisfaction according to the SWLS, adherence according to the ARMS, and acceptance of illness according to the AIS.

Based on the results, it was observed that life satisfaction according to the SWLS was only affected by emotion-oriented coping. Patients using this style of coping with stress to a greater extent were characterized by significantly lower life satisfaction than other patients (β_std._ = -0.445, p < 0.001). There were no statistically significant relationships between the other stress coping styles according to the CISS and life satisfaction (**Table 3**).

**Table 3:** The effect of a stress coping style according to the CISS on the variables studied.

	Stress coping style (sten scores)						
	Intercept		TOC	EOC	AOC	Distraction	Social diversion
SWLS	b	23.497	0.088	-1.277	0.451	-0.745	0.737
	βstand.		0.026	-0.445	0.134	-0.21	0.26
	p	<0.001	0.789	<0.001	0.603	0.266	0.153
ARMS	b	15.829	-0.103	-0.192	-0.508	1.544	-0.595
	βstand.		-0.036	-0.079	-0.179	0.517	-0.249
	p	<0.001	0.739	0.43	0.531	0.015	0.217
AIS	b	35.217	0.471	-1.786	2.054	-1.608	-0.52
	βstand.		0.116	-0.517	0.507	-0.377	-0.153
	p	<0.001	0.25	<0.001	0.058	0.054	0.414

TOC, task-oriented coping; EOC, emotion-oriented coping; AOC, avoidance-oriented coping; distraction, engaging in alternative activities); social diversion (seeking social contact); SWLS, Satisfaction with Life Scale; ARMS, Adherence to Refills and Medication Scale; AIS, Acceptance of Illness Scale; b, regression coefficient; β_stand._, standardized regression coefficient.

The stress coping style in the form of distraction (engaging in alternative activities) was found to significantly affect the level of adherence in chronic diseases according to the ARMS. Patients who used this style to a greater extent scored significantly higher on the adherence scale (worse adherence to therapeutic recommendations) than the rest of the respondents (β_std._ = 0.517, p = 0.015). There were no statistically significant relationships between the other stress coping styles according to the CISS and the level of adherence (**Table 3**).

Based on the collected data, emotion-oriented coping had a statistically significant impact on the level of illness acceptance according to the AIS. Patients who used this style to a greater extent were characterized by significantly worse acceptance of the disease than the rest of the respondents (β_std._ = -0.517, p < 0.001). There were no statistically significant relationships between the other stress coping styles according to the CISS and acceptance of the disease (**Table 3**).

The study analyzed the impact of a stress coping style according to the CISS on QoL according to the SF-36.

Task-oriented coping was found to have an impact on QoL. It was related to the domains of role physical (RP) (β_std._ = 0.237, p = 0.042) and mental health (MH) (β_std._ = 0.287, p = 0.001), as well as to physical component summary (PCS) (β_std._ = 0.228, p = 0.042) and mental component summary (MCS) (β_std._ = 0.188, p = 0.034). There were no statistically significant relationships with the other SF-36 domains (**Table 4**).

**Table 4:** The impact of a stress coping style according to the CISS on QoL according to the SF 36.

	Stress coping styles (sten scores)						
	Intercept		TOC	EOC	AOC	Distraction	Social diversion
Role physical (RP)	b	69.932	3.034	-2.287	1.779	-2.642	-1.052
	βstand.		0.237	-0.209	0.139	-0.196	-0.098
	p	<0.001	0.042	0.052	0.647	0.379	0.648
General health (GH)	b	52.084	0.794	-2.68	1.699	-2.054	1.081
	βstand.		0.115	-0.454	0.245	-0.282	0.185
	p	<0.001	0.226	<0.001	0.324	0.123	0.29
Social functioning (SF)	b	57.938	1.832	-3.01	2.453	-3.711	2.81
	βstand.		0.137	-0.264	0.184	-0.264	0.25
	p	<0.001	0.184	0.006	0.498	0.184	0.191
Role emotional (RE)	b	69.026	1.361	-3.176	3.188	-4.314	1.154
	βstand.		0.099	-0.271	0.232	-0.298	0.1
	p	<0.001	0.369	0.009	0.424	0.161	0.624
Vitality (VT)	b	60.61	0.77	-3.355	3.321	-3.754	1.033
	βstand.		0.094	-0.481	0.406	-0.436	0.15
	p	<0.001	0.28	<0.001	0.079	0.011	0.353
Mental health (MH)	b	58.586	2.238	-3.381	-2.272	0.53	2.211
	βstand.		0.287	-0.508	-0.291	0.065	0.336
	p	<0.001	0.001	<0.001	0.19	0.69	0.033
Physical component summary (PCS)	b	72.755	1.688	-1.392	1.241	-1.76	0.361
	βstand.		0.228	-0.22	0.168	-0.226	0.058
	p	<0.001	0.042	0.034	0.566	0.292	0.778
Mental component summary (MCS)	b	61.057	1.575	-3.29	1.038	-2.204	1.727
	βstand.		0.188	-0.46	0.124	-0.25	0.245
	p	<0.001	0.034	<0.001	0.592	0.142	0.134

TOC, task-oriented coping; EOC, emotion-oriented coping; AOC, avoidance-oriented coping; distraction, engaging in alternative activities; social diversion, seeking social contact; b, regression coefficient; β_stand._, standardized regression coefficient.

The results of the study showed that QoL was also affected by an emotion-oriented coping style. This style was linked to the domains of general health (GH) (β_std._ = -0.454, p < 0.001), social functioning (SF) (β_std._ = -0.264, p = 0.006), role emotional (RE) (β_std._ = -0.271, p = 0.009), vitality (VT) (β_std._ = -0.481, p < 0.001), and mental health (MH) (β_std._ = -0.508, p < 0.001), as well as to physical component summary (PCS) (β_std._ = -0.220, p = 0.034) and mental component summary (MCS) (β_std._ = -0.460, p < 0.001). There were no statistically significant relationships with the other SF-36 domains.

Distraction (engaging in alternative activities) had an impact on vitality (VT) (β_std._ = -0.436, p = 0.011). Respondents who made greater use of this coping style had a significantly lower QoL than those who made less use of it. There were no statistically significant relationships between engaging in alternative activities and the other SF-36 domains.

Social diversion (seeking social contact) had an impact on mental health (MH) (β_std._ = 0.336, p = 0.033). Patients who made greater use of this style had significantly higher QoL than those who used it to a lesser extent (**Table 4**). There were no statistically significant relationships between seeking social contact and the other SF-36 domains.

Avoidance-oriented coping had no statistically significant effect on any of the SF-36 domains (**Table 4**).

## 4 Discussion

Once an accurate diagnosis is provided and appropriate medications are prescribed, treatment adherence plays an important role in achieving good health outcomes and maintaining good quality of life in patients with mood disorders. Non-adherence is a barrier to effective treatment.

A review of the literature indicates that there are few publications on the effects of stress coping styles on treatment adherence, life satisfaction, acceptance of a disease, and QoL among people with mood disorders. Our study is probably the first to assess the impact of stress coping styles on psychological variables in this population.

In our study, the main stress coping style significantly affecting the examined psychological variables was emotion-oriented coping. It was found to influence acceptance of a disease, QoL, and life satisfaction. Patients who made greater use of this style were characterized by significantly lower acceptance of the disease and lower life satisfaction. Poor QoL was mainly noted in the dimensions of general health (GH), social functioning (SF), role emotional (RE), vitality (VT), mental health (MH), and physical function (PF), as well as in physical component summary (PCS) and mental component summary (MCS). We also observed that distraction (engaging in alternative activities) had a significant effect on vitality (VT) and adherence in chronic diseases. Patients who used this style to a greater extent achieved substantially higher scores for adherence, and had significantly lower QoL in the vitality (VT) domain than the rest of the respondents. Task-oriented coping affected the domains of role physical (RP), mental health (MH), as well as physical component summary (PCS) and mental component summary (MCS). Seeking social contact, on the other hand, is related to the domain of mental health (MH).

A study by Szeliga-Lewinska and Landowki showed that people from the clinical group were characterized mainly by an emotion-oriented style, while the general population more often used task-oriented coping. In terms of personality traits, people with depression had higher levels of neuroticism, while the level of extraversion was higher in the control population (33). Similar results were obtained by Borowiecka-Karpiuk et al. in their study of people with depressive disorders or bipolar disorder, the dominant stress coping style was focused on emotions (34). McWilliams et al. found that emotion-oriented coping was associated with depression (35). Suh et al. used the CISS and the Beck Depression Inventory (BDI) to analyze similarities and differences in stress coping strategies in a group of 135 people with bipolar disorder and 100 people with depressive disorders. They found that the severity of depressive symptoms can affect the style of coping with stress, therefore the authors divided the group with bipolar disorder into patients with and without depressive symptoms, and the group with depression into patients with and without exacerbation of symptoms. Thanks to this division, it was noted that patients with bipolar disorder and depressive symptoms were less likely to use task-oriented coping and avoidance-oriented coping, and more likely to apply emotion-oriented coping than bipolar patients without depression (36). A review of the literature indicates that many people with mood disorders choose maladaptive methods of coping with the disease (37–41).

Alemayehu et al. (42) demonstrated that poor QoL of patients with depressive disorders was significantly linked to their non-adherence to treatment recommendations. Similar results were obtained in a study conducted as part of the EU National Health and Wellbeing Survey (NHWS) (43). The likely reasons for this phenomenon may be the fact that patients with chronic conditions (including depression) do not adhere to treatment due to social, physical and mental health problems, which are indicators of poor QoL (44, 45).

Pacian et al. (46) found no statistically significant differences between the risk of depression and overall QoL. It has been observed that with older age, depression becomes more common and QoL in the mental and social domains decreases. Makara-Studzińska et al. (47), on the other hand, confirmed a significant relationship between the severity of depression and QoL in the physical domain in schizophrenic patients, and Daly et al. (48) informed that depressive disorders negatively affected QoL.

Kelly et al. (49) provided evidence that people with mood disorders showed more emotional reactions, which was associated with choosing maladaptive coping styles by both women and men. Furthermore, in women, greater control over depression was related to more adaptive coping strategies.

## 5 Conclusions

Treatment non-adherence is a serious challenge in the treatment of patients with mood disorders. Individuals who do not adequately follow treatment recommendations often resort to alternative activities as a mechanism for coping with difficult situations. Patients who predominantly adopt an emotion-oriented coping style tend to experience lower life satisfaction and greater difficulty accepting their condition compared to their peers. Conversely, patients who adopt a task-oriented coping style report better QoL than those who rely on emotion-oriented coping or alternative activities. Additionally, patients who actively seek out social contacts tend to enjoy a significantly higher QoL than those who are less likely to use this coping style.

## 6 Limitation

Our study has some limitations. Most data were measured using self-report questionnaires, which may have led to biased reporting of experiences. Our study is cross-sectional, so the current study is unable to demonstrate a temporal causal relationship between treatment non-adherence and the main potential contributors. Another limitation pertains to the sample size. The Polish sample was not representative, which limits the ability to generalize the results to other populations.

## Data availability statement

The raw data supporting the conclusions of this article will be made available by the authors, without undue reservation.

## Ethics statement

The studies involving humans were approved by Bioethics Committee of the Pomeranian Medical University in Szczecin (KB-0012/153/17). The studies were conducted in accordance with the local legislation and institutional requirements. The participants provided their written informed consent to participate in this study.

## Author contributions

AJ: Writing – review & editing, Project administration, Methodology, Formal analysis, Conceptualization. DS-M: Writing – review & editing, Validation, Software. KR: Writing – review & editing, Investigation, Formal analysis, Data curation. AR: Writing – review & editing, Formal analysis. MP: Writing – review & editing, Data curation. ĆD: Writing – review & editing, Supervision. EG: Writing – review & editing, Resources, Funding acquisition. AC: Writing – review & editing, Writing – original draft, Visualization, Methodology, Conceptualization.
